# Theoretical Models and Simulations of Gene Delivery with Polyurethane: The Importance of Polyurethane as a Vector in Personalized Therapy

**DOI:** 10.3390/biomedicines13030692

**Published:** 2025-03-11

**Authors:** Roxana Maria Jeleriu, Roxana-Karin Hajaj, Iuliana-Anamaria Trăilă, Mihaela Zaharie, Maria Puiu

**Affiliations:** 1 Ph.D. School, Faculty of Medicine, Department of Microscopic Morphology, Genetics Discipline, Center of Genomic Medicine, “Victor Babes” University of Medicine and Pharmacy Timisoara, 2 E. Murgu, Sq., 300041 Timisoara, Romania; roxana.jeleriu@umft.ro (R.M.J.); roxana.ianes@umft.ro (R.-K.H.); maria_puiu@umft.ro (M.P.); 2 Department of Pediatric Surgery and Orthopedics, “Louis Țurcanu” Emergency Clinical Hospital for Children, 300011 Timisoara, Romania; 3 Department of Pathology, ‘Pius Brinzeu’ Emergency County Clinical Hospital, 300723 Timisoara, Romania; 4 Department XII Obstetrics-Gynecology, ‘Victor Babeş’ University of Medicine and Pharmacy of Timișoara, 300041 Timisoara, Romania; mihaela.zaharie@umft.ro; 5 Neonatology-Premature Unit, “Louis Țurcanu” Emergency Clinical Hospital for Children, 300011 Timisoara, Romania; 6 Institute for Research and Development in Genomics, 020021 București, Romania

**Keywords:** gene delivery, theoretical model, polyurethane vectors, simulations, personalized therapy, drug delivery systems, nanomedicine

## Abstract

**Background/Objectives:** Advancements in personalized medicine have revolutionized drug delivery, enabling tailored treatments based on genetic and molecular profiles. Non-viral vectors, such as polyurethane (PU)-based systems, offer promising alternatives for gene therapy. This study develops mathematical models to analyze PU degradation, DNA/RNA release kinetics, and cellular interactions, optimizing their application in personalized therapy. **Methods**: This theoretical study utilized mathematical modeling and numerical simulations to analyze PU-based gene delivery, focusing on diffusion, degradation, and cellular uptake. Implemented in Python 3.9, it employed differential equation solvers and adsorption/internalization models to predict vector behavior and optimize delivery efficiency. **Results**: This study demonstrated that PU degrades in biological environments following first-order kinetics, ensuring a controlled and predictable release of genetic material. The Higuchi diffusion model confirmed a gradual, sustained DNA/RNA release, essential for efficient gene delivery. Simulations of PU adsorption onto cellular membranes using the Langmuir model showed saturation-dependent binding, while the endocytosis model revealed a balance between uptake and degradation. These findings highlight PU’s potential as a versatile gene delivery vector, offering controlled biodegradability, optimized release profiles, and effective cellular interaction. **Conclusions**: Our results confirm that PU-based vectors enable controlled biodegradability, sustained DNA/RNA release, and efficient cellular uptake. Mathematical modeling provides a framework for improving PU’s properties, enhancing transport efficiency and therapeutic potential in personalized medicine and gene therapy applications.

## 1. Introduction

The foundation of personalized medicine lies in the understanding that individual patients exhibit varying responses to the same treatment due to genetic differences [1]. Personalized drug therapy, also known as precision medicine, signifies a revolutionary shift in healthcare, customizing medical treatments to align with the unique characteristics of each patient. This transformation is propelled by advancements in genomics, pharmacogenomics, and a more profound comprehension of disease mechanisms, enabling healthcare providers to tailor therapeutic strategies according to a patient’s genetic profile and environmental influences [2]. While the impact of personalized medicine has been most notable in oncology, its principles are increasingly being adopted across diverse medical fields, including psychiatry, cardiology, endocrinology, and genetic syndromes [2].

Regulatory frameworks that promote the inclusion of genetic testing in drug labeling and prescribing guidelines have supported the integration of pharmacogenomics into clinical practice [3]. The U.S. Food and Drug Administration (FDA) has acknowledged the significance of pharmacogenomic information, with over 160 medications now incorporating such data in their package inserts [4,5].

The application of computational biology and data mining techniques has also emerged as a promising avenue for advancing personalized drug therapy. By leveraging large datasets and sophisticated algorithms, researchers can identify new drug indications and optimize therapeutic strategies based on genetic and molecular profiles [6]. As personalized medicine continues to evolve, integrating artificial intelligence and machine learning into drug development processes holds significant promise. The potential of AI-driven insights to enhance personalized drug therapy is particularly relevant in complex diseases [7].

Gene therapy is an essential component of personalized therapies. It is based on genetic analysis of the patient and the correction of specific mutations based on genomics and proteomics and other biological information data. This therapeutic approach generally encompasses multiple strategies, such as deactivating a gene responsible for causing disease, replacing a defective gene with a functional one, or introducing novel or altered genes to fight illnesses [8]. The potential advantages of gene therapy are significant, with particular applications resulting in complete cures for conditions that were once considered untreatable [8].

A key factor in the success of gene therapy is the efficient delivery of genetic material to the targeted cells. Various delivery systems, including viral and non-viral vectors, have been developed to ensure therapeutic genes reach their intended destinations within the body [9]. Viral vectors, such as adenoviruses and lentiviruses, have demonstrated high efficiency in gene delivery due to their natural capacity to infect host cells. However, concerns related to immunogenicity and the risk of insertional mutagenesis have led researchers to investigate non-viral delivery methods, such as lipid-based nanoparticles (liposomes, cationic lipids, lipoplexes), polymeric carriers (polyethyleneimine (PEI), poly(L-lysine) (PLL), polyurethane (PU), chitosan), peptide-based vectors (cell-penetrating peptides (CPPs), protamine–DNA complexes), inorganic nanoparticle-based vectors (gold nanoparticles (AuNPs), silica nanoparticles, magnetic nanoparticles (MNPs)), and hybrid systems (lipid–polymer hybrid nanoparticles, liposome–protein conjugates), which provide safer options [9,10]. These advancements in vector technology are essential for improving the effectiveness and safety of gene therapy applications.

Thanks to their unique properties that enable the efficient delivery of genetic material, PU-based vectors have gained attention as a promising non-viral option for gene therapy. These polymers can be tailored to exhibit specific traits, such as biocompatibility, biodegradability, and the ability to form nanoparticles capable of encapsulating nucleic acids [11]. Their versatility allows for modifications that enhance cellular uptake, endosomal escape, and targeted delivery, making them ideal for a wide range of therapeutic applications [12,13,14].

Conversely, PUs are a class of versatile polymers with extensive applications across various industries beyond the medical field. Their unique properties, including durability, flexibility, and chemical resistance, make them suitable for diverse applications in textiles, automotive, construction, and electronics. In the textile industry, PU coatings can enhance the performance of textile products by providing properties such as water repellency, UV resistance, and mechanical durability [15,16]. PU foams are particularly valued for their lightweight yet strong characteristics, making them ideal for vehicle interiors, such as seat cushioning and insulation, in the automotive industry [17]. Moreover, due to their excellent resilience and durability, polyurethane elastomers are employed in tires, gaskets, and O-rings [17]. The construction industry also benefits significantly from polyurethane technologies. PU foams are insulation materials due to their superior thermal resistance properties [18]. They are used in flexible electronics coatings, adhesives, and elastomers, offering strong adhesion, environmental resistance, and eco-friendly alternatives to solvent-based systems [19,20,21]. In the paint and coatings sector, their exceptional adhesion, hardness, and chemical resistance make them ideal for high-performance applications, such as automotive finishes and protective coatings. At the same time, flame-retardant formulations enhance safety in demanding environments [17,22,23]. Polyurethane nanocomposites reinforced with nanoparticles exhibit improved mechanical and thermal properties, expanding their use in structural materials and coatings [22].

A key advantage of PU vectors is their ability to self-assemble into nanoparticles that encapsulate DNA or RNA. This feature is essential for protecting nucleic acids from degradation in biological environments and ensuring their efficient delivery to target cells [13,24,25]. Additionally, by incorporating targeting ligands on their surface, these nanoparticles can facilitate receptor-mediated endocytosis, improving the precision of gene delivery to specific cell types [26]. This targeted approach is especially valuable in cancer therapy, where delivering therapeutic genes directly to tumor cells can enhance treatment outcomes while minimizing harm to healthy tissues [14]. Recent advancements have also highlighted the potential of biodegradable PU as gene carriers [27,28]. These materials break down into non-toxic byproducts, reducing the risk of long-term accumulation in the body and associated adverse effects [29]. For example, an L-tyrosine-based PU was developed as a biocompatible and biodegradable polymer, demonstrating high transfection efficiency with minimal toxicity [29]. The ability to design PUs that degrade in response to specific physiological conditions, such as pH changes, further enhances their utility in gene therapy [26].

PUs face several limitations. In biomedical applications, challenges related to biocompatibility and bioactivity can be overcome by functionalizing PUs with bioactive compounds or peptides or through surface modifications like plasma treatment to enhance hydrophilicity. For drug delivery systems, issues such as burst release or insufficient sustained release can be addressed by developing composite structures (e.g., core-shell configurations) or stimuli-responsive designs triggered by pH or temperature changes. Crosslinking agents can also help regulate drug release rates [30].

PU vectors are not limited to cancer therapy; they are also being explored for other applications, including cardiovascular diseases and genetic disorders.

Despite their potential, optimizing PU vectors for clinical use remains challenging. Scalable production, reproducibility, and comprehensive safety evaluations must be addressed to transition from laboratory research to clinical practice [31]. Furthermore, the regulatory framework for gene therapy products is complex, requiring detailed characterization and validation of PU-based systems before they can be approved for human use. As a result, these vectors are still in the experimental testing phase and have not yet been approved for human treatments [32].

This theoretical study aims to develop mathematical models and numerical simulations that describe the behavior of a PU-based gene delivery system. The research focuses on three key aspects: the diffusion and degradation of PU in biological environments, the kinetics of DNA/RNA release from a PU-based system, and the interaction of PU vectors with cellular membranes.

These models are designed to understand the transport mechanisms better and optimize gene delivery efficiency. By exploring these factors, the study aims to contribute to advancing personalized therapy by using PU as a versatile and practical vector.

The results were obtained by solving first-order kinetic equations and employing numerical simulations for diffusion and reaction mechanisms. All graphical representations were generated in Python 3.9, ensuring precise and reproducible data visualization. This computational approach allows for a predictive understanding of PU’s performance in gene therapy without needing laboratory experimentation, serving as a preliminary theoretical framework for future experimental validation.

## 2. Materials and Methods

This study, conducted in January 2025, was designed as a purely theoretical approach to modeling the behavior of polyurethane (PU) as a gene delivery vector. Given the computational and non-experimental nature of the research, no biological samples, in vitro experiments, or in vivo procedures were required. Consequently, approval from the Ethics Committee of the Doctoral School was not necessary. The study was based on mathematical modeling and numerical simulations, utilizing computational tools to analyze and predict PU-based drug and gene delivery systems’ diffusion, degradation, and cellular uptake.

### 2.1. Computational Framework and Tools

The research employed theoretical frameworks, mathematical equations, and computational algorithms implemented in Python 3.9. Key Python libraries used included NumPy for numerical operations, SciPy for solving differential equations (specifically scipy.integrate.odeint), and Matplotlib version 3.10.1, for data visualization. These tools were selected for their robustness and efficiency in handling complex mathematical models and simulations.

### 2.2. Simulation Design

The simulations focused on three critical processes:

- Diffusion and degradation of PU in a biological medium: Mathematical models were developed to describe the diffusion kinetics of PU and its degradation in a simulated biological environment.
- Controlled release kinetics of genetic material: The study utilized differential equations to model the release profiles of DNA/RNA from PU-based systems, ensuring controlled and sustained delivery.
- Interaction of PU vectors with cellular membranes: The Langmuir adsorption model was applied to simulate the binding of PU vectors to cellular membranes. Additionally, an internalization model incorporating uptake and intracellular degradation rates was used to predict the absorption and fate of PU vectors within cells.

### 2.3. Materials and Theoretical Models

The primary materials used in this research were theoretical frameworks and mathematical models, including:

- Degradation Kinetics Models: To describe the breakdown of PU under simulated physiological conditions.
- Controlled Release Models: To predict the release rates of genetic material from PU-based systems.
- Adsorption and Internalization Models: To simulate the interaction of PU vectors with cellular membranes and their subsequent internalization.

### 2.4. Selection of Materials and Parameters for PU–DNA Interaction

To simulate the interaction between PU and DNA, the following parameters and assumptions were used:

1. Polyurethane (PU) Properties:
  - Degradation Rate Constant (k): The degradation rate constant was set based on previous studies of biodegradable polymers, with values ranging from 10^−3^ to 10^−2^ h^−1^, depending on the simulated biological environment (e.g., pH, temperature) [33].
  - Diffusion Coefficient (D): The diffusion coefficient of PU in a biological medium was estimated using the Stokes–Einstein equation. Depending on the medium’s molecular weight and viscosity, the value typically ranges from 10^−12^ to 10^−10^ m^2^/s [33].
  - Initial Concentration (C0): Based on typical concentrations used in drug delivery studies, the initial concentration of PU was set to 1 mg/mL [33].

2. DNA Properties:
  - Molecular Weight: The DNA used in the simulations was assumed to have a molecular weight of 5 kDa, representing a typical plasmid DNA size [34].
  - Charge Density: DNA was modeled as a negatively charged molecule, with a charge density of −1 × 10^0^ e/base pair, consistent with its phosphate backbone [35].
  - Encapsulation Efficiency: Based on previous studies of polymeric gene delivery systems, the encapsulation efficiency of DNA within the PU matrix was assumed to be 90% [35].

3. PU–DNA Interaction Parameters:
  - Binding Affinity (K): The binding affinity between PU and DNA was modeled using electrostatic interactions, with a binding constant *K* set to 10^5^ M^−1^, based on literature values for cationic polymers interacting with nucleic acids [36].
  - Release Rate Constant (krel): The release rate constant for DNA from the PU matrix was set to 10^−3^ h^−1^, ensuring a controlled and sustained release profile [36].
  - Diffusion Length (L): The diffusion length of DNA through the PU matrix was set to 100 nm, based on the typical size of PU nanoparticles used in gene delivery [36].

4. Cellular Uptake Parameters:
  - Adsorption Constant (Kads): Based on the Langmuir adsorption model, the adsorption constant for PU nanoparticles on cellular membranes was set to 10^6^ M^−1^ [37].
  - Uptake Rate Constant (kuptake): The internalization rate of PU vectors via endocytosis was set to 10^−2^ h^−1^, consistent with previous studies of nanoparticle-mediated gene delivery [37].
  - Degradation Rate Constant (kdegr): The intracellular degradation rate of PU vectors was set to 10^−3^ h^−1^, ensuring a balance between uptake and degradation [37].

This study focused exclusively on computational modeling, employing mathematical simulations and theoretical frameworks to analyze the behavior of polyurethane-based gene delivery systems. The methodology was organized into distinct subsections, detailing the computational tools, simulation design, and theoretical models. By leveraging advanced numerical techniques and Python-based libraries, the research provided a comprehensive theoretical analysis of PU as a gene delivery vector, without the need for experimental validation.

Following COPE guidelines [38], this study utilized AI-assisted tools to enhance manuscript preparation. Specifically, ChatGPT (OpenAI, Version 4.0) and Grammarly (Grammarly Inc. San Francisco, CA, USA, Premium Edition) were employed during the writing, editing, and graphical visualization processes. These tools were used between 12 January 2025 and 24 January 2025 to refine language clarity, improve grammatical structure, and generate conceptual illustrations related to polyurethane-based gene delivery. The authors confirm that they take full responsibility for the integrity and accuracy of the content generated. They have critically reviewed all AI-assisted outputs to ensure compliance with scientific rigor and ethical standards.

## 3. Results

### 3.1. Assumptions and Basic Equation

Gene delivery involves encapsulating DNA/RNA within a PU matrix, followed by its controlled release. This process can be described using a controlled release model:

(1) $$\frac{dM}{dt}=-k_{rel}M$$

where:

- *M*(*t*) represents the remaining mass of DNA/RNA within the PU matrix at time t.
- *k_rel_* is the release rate constant, which depends on environmental factors such as pH and temperature.

This equation provides a foundational framework for understanding DNA/RNA release kinetics from the PU matrix.

The initial mass of encapsulated DNA/RNA (M_0_) was set to 1 mg, representing a typical loading capacity for PU-based gene delivery systems [39].

**Figure 1:**
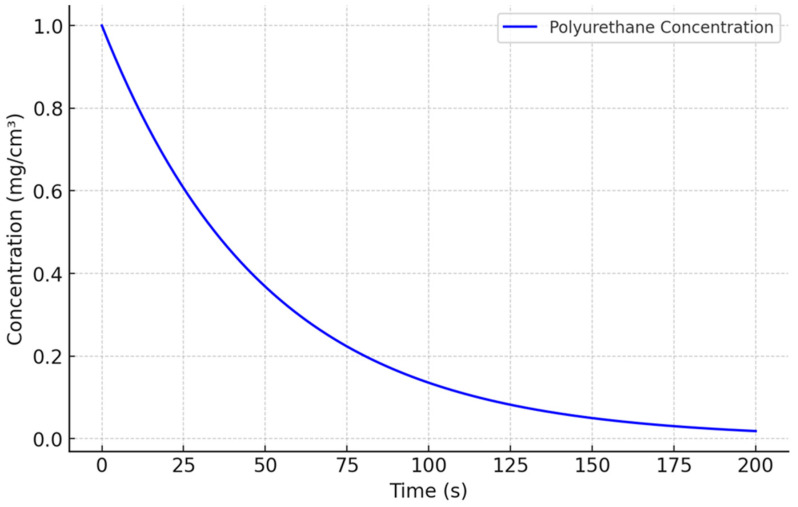
PU degradation in biological medium.

We conducted a simulation to analyze the degradation of PU in a biological environment. The results are presented in Figure 1, illustrating a decrease in PU concentration over time following first-order kinetics:

(2) $$C(t)=C_{0}e^{-kt}$$

where:

- *C*(*t*) represents the concentration of PU at time t.
- *C*_0_ is the initial concentration of PU.
- *k* is the degradation rate constant, which depends on the specific biological conditions.

Figure 1 demonstrates a gradual reduction in PU concentration, indicating a progressive degradation process. This behavior aligns with the expected first-order kinetics, where the degradation rate is proportional to the remaining concentration of the material. The results highlight the ability of PU to degrade steadily in biological environments, which is a critical factor for its application in controlled gene delivery systems. This degradation profile provides valuable insights into the design of PU-based carriers, ensuring their compatibility with biological systems and ability to release genetic material in a controlled and predictable manner.

### 3.2. Higuchi Model for Diffusion

A more advanced model considers the diffusion-controlled release of DNA/RNA through the PU structure utilizing the Higuchi mode:

(3) $$M_{t}=M_{0}\left(1-\sqrt{\frac{Dt}{L^{2}}}\right)$$

where:

- *M_t_* is the mass of DNA/RNA released at time *t*.
- *M*_0_ is the initial mass of encapsulated DNA/RNA.
- *D* is the diffusion coefficient, which characterizes the diffusion rate through the PU matrix.
- *L* is the length of the PU matrix.
- The initial concentration of DNA/RNA within the PU matrix was set to 1 mg/mL, consistent with experimental conditions used in previous studies [40].

The Higuchi model allows for simulating the release profile of genetic material and provides a basis for optimizing parameters to achieve efficient gene delivery. By incorporating these models, the study aimed to enhance the understanding of release kinetics and improve the design of PU-based gene delivery systems.

**Figure 2:**
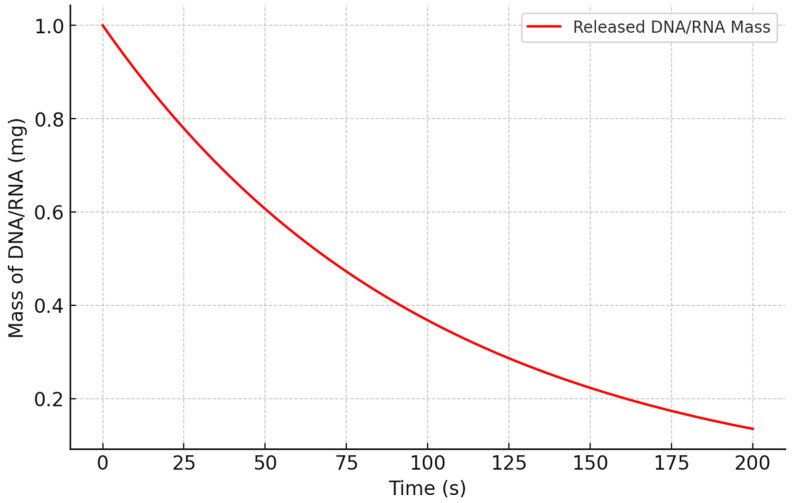
Release of DNA/RNA from PU matrix.

We conducted simulations to analyze the release kinetics of DNA/RNA from a PU matrix. The results are illustrated in Figure 2, which depicts an exponential decrease in the mass of DNA/RNA over time. This trend indicates a gradual and controlled release of the genetic material from the PU matrix. Such behavior is consistent with the release profiles typically observed in biodegradable polymer systems, where the polymer matrix’s degradation governs the release rate.

The exponential decay observed in the simulation aligns with the theoretical model of controlled release, confirming that the PU matrix effectively modulates the delivery of DNA/RNA. This controlled release mechanism is fundamental for ensuring sustained and efficient gene delivery, as it minimizes premature release and maximizes the therapeutic impact of the genetic material. These findings highlight the potential of PU as a versatile and reliable vector for gene delivery applications.

### 3.3. Modeling the Interaction of PU Vectors with Cellular Membranes

#### 3.3.1. Assumptions

The interaction between PU-based vectors and cells can be described through the adsorption and internalization of nanoparticles. Several key factors influence this process:

- The electric charge of the PU.
- The type of cellular receptors present on the membrane.
- The dynamics of the lipid bilayer of the cellular membrane.

These factors collectively determine the efficiency of cellular uptake and the subsequent delivery of genetic material.

#### 3.3.2. Modeling PU Adsorption on the Cellular Membrane

The adsorption of PU nanoparticles onto the cellular membrane can be modeled using the Langmuir adsorption model:

(4) $$\theta =\frac{KC}{1+KC}$$

where:

- *θ* represents the fraction of the membrane surface covered by PU nanoparticles.
- *K* is the adsorption constant, which depends on the nanoparticle and membrane affinity.
- *C* is the concentration of nanoparticles in the extracellular environment.

This model provides a quantitative framework for understanding how PU nanoparticles adhere to the cell surface, a critical step in gene delivery.

The initial concentration of PU nanoparticles in the extracellular environment was set to 1 mg/mL, consistent with experimental conditions [41].

**Figure 3:**
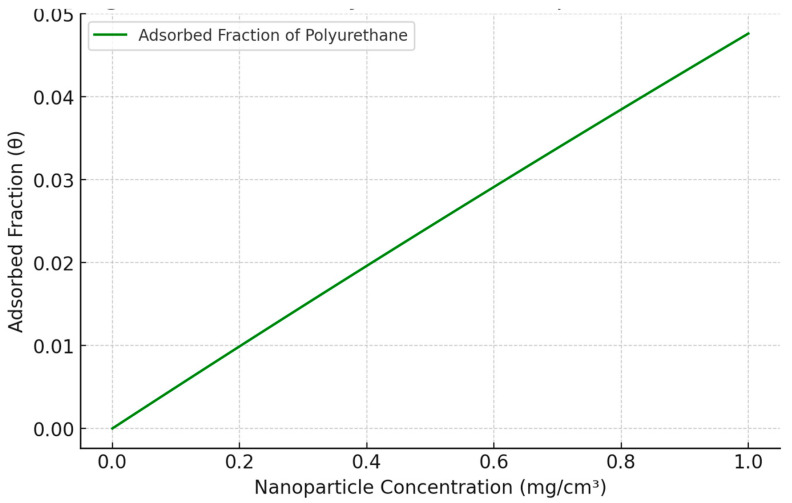
Langmuir model for PU adsorption on membrane.

The relationship between nanoparticle concentration and the fraction of surface coverage, as predicted by the Langmuir model, is illustrated in Figure 3. The plot demonstrates a rapid increase in surface coverage followed by a plateau, indicating a limited number of binding sites on the cellular membrane. This saturation behavior is characteristic of adsorption processes governed by the Langmuir model and highlights the finite capacity of the membrane to interact with PU nanoparticles.

#### 3.3.3. Modeling Internalization via Endocytosis

The internalization of PU vectors into the cell can be described using a differential equation that accounts for the uptake and degradation processes:

(5) $$\frac{dN}{dt}=k_{uptake}\theta N_{max}-k_{degr}N$$

where:

- *N*(*t*) is the number of internalized PU vectors at time *t*.
- $k_{uptake}$ is the rate of internalization, which depends on the efficiency of endocytosis.
- $N_{max}$ is the maximum number of available binding sites on the cellular membrane.
- $k_{degr}$ is the rate of degradation of the PU vectors within the cell.

The initial number of internalized PU vectors (*N*_0_) was set to 0, as no internalization occurs at *t* = 0.

This equation allows for simulation of the dynamics of PU vector uptake and provides insights into optimizing the design of these vectors for enhanced gene delivery efficiency. By integrating these models, the study aimed to improve understanding of the interactions between PU-based vectors and cellular membranes, ultimately contributing to developing more effective gene delivery systems.

**Figure 4:**
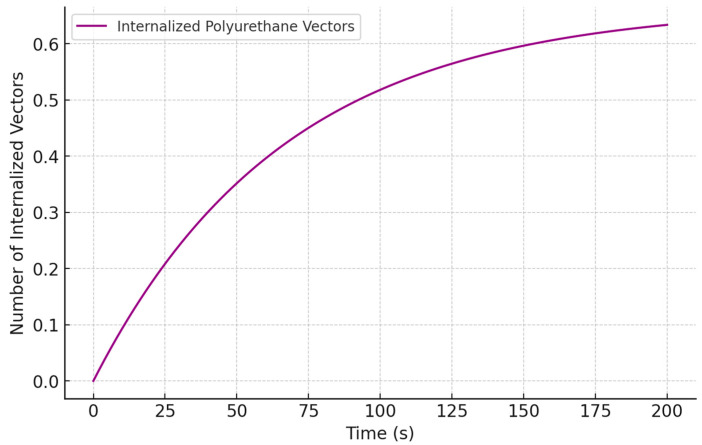
Internalization of PU vectors in cells.

Figure 4 illustrates the progressive increase in the number of PU vectors internalized by cells. Once a specific intracellular concentration is reached, the internalization rate slows due to intracellular degradation and the limited availability of binding sites on the cellular membrane. This behavior highlights the dynamic balance between uptake and degradation processes, which is critical for optimizing the efficiency of gene delivery systems.

## 4. Discussion

Incorporating theoretical models and simulations into the study of gene delivery systems has yielded critical insights into the mechanisms and optimization of PU-based vectors. As a highly adaptable and promising carrier, PU is central to driving progress in personalized therapy, providing customized solutions for efficient and precise gene delivery. The effectiveness of PU can be significantly improved by utilizing it in nanoparticle systems, owing to their superior water solubility, enhanced drug distribution, increased biocompatibility, and precise targeting capabilities [24]. This discussion delves into the importance of PU as a vector, highlighting its distinctive properties, the contribution of computational methods in elucidating its behavior, and its transformative potential in advancing gene therapy within the precision medicine framework.

The existing research on PU as a gene delivery vector remains relatively scarce. Only a handful of studies have explored this complex system, which holds potential as a delivery mechanism for personalized medicine therapies [13,25,26,27]. Instead, most research efforts have concentrated on developing PU nanoparticles as carriers [28,29,30,31,32].

PUs’ versatility enables modifications that enhance cellular uptake, endosomal escape, and targeted delivery, making them highly suitable for various therapeutic applications [13,25]. Studies have shown that one of the key advantages of PU vectors in gene therapy is their ability to self-assemble into nanoparticles capable of encapsulating DNA or RNA. This self-assembly property is critical for protecting nucleic acids from degradation in biological environments and ensuring their efficient transport into target cells [13].

The results of our study align with existing literature on PU-based gene delivery systems, reinforcing the material’s potential as a controlled-release vector [16,42,43,44]. The observed degradation pattern of PU in a biological medium follows first-order kinetics, consistent with previous research highlighting the predictable breakdown of biodegradable polymers in physiological environments. This gradual degradation is essential for sustained gene release, ensuring that DNA/RNA remains protected within the matrix while being delivered at a controlled rate. These properties make PU a promising candidate for gene therapy applications.

Research highlights that cationic PUs, in particular, are effective at forming stable complexes with nucleic acids, significantly improving transfection efficiency. The electrostatic interactions between the positively charged polymer and negatively charged nucleic acids are central to creating these complexes, enabling effective cellular uptake [25].

Further research has revealed that incorporating trialkyl phosphonium groups into cationic PUs can enhance gene delivery capabilities. These modifications improve nanoparticle stability and enable the controlled release of encapsulated DNA, which is essential for achieving optimal transfection rates [25].

The results from our study demonstrate that the PU matrix enables a controlled and sustained release of DNA/RNA, aligning with the diffusion-based Higuchi model. This release profile, characterized by an exponential decrease in genetic material over time, is consistent with findings in biodegradable polymer systems, where degradation plays a key role in modulating drug and gene delivery. The literature has reports of similar trends, particularly in studies exploring PU modifications for enhanced gene transfection efficiency [33,34,35].

The literature highlights that the design of PU vectors can be adapted to overcome specific challenges in gene delivery. For example, modifications can enhance the stability of polyplexes during formulation and application, ensuring the integrity of genetic material throughout the delivery process [13]. Additionally, incorporating targeting ligands onto the surface of PU nanoparticles facilitates receptor-mediated endocytosis, improving the specificity of gene delivery to particular cell types [13].

Our research demonstrates that the interaction between PU-based vectors and cellular membranes follows the well-established adsorption and internalization principles observed in nanoparticle-mediated gene delivery. The Langmuir adsorption model effectively describes how PU nanoparticles adhere to the cell membrane, revealing a saturation point where available binding sites become fully occupied. This aligns with previous findings in the literature, which emphasize the importance of nanoparticle surface properties, such as charge and functionalization, in optimizing membrane interactions. Furthermore, the internalization model highlights the dynamic equilibrium between uptake and degradation, mirroring studies that propose modifications to enhance vector stability and cellular targeting.

Recent studies have demonstrated the potential of biodegradable PUs as gene carriers. These materials degrade into non-toxic byproducts, reducing the risk of long-term accumulation in the body and associated adverse effects [25]. For instance, an L-tyrosine-based PU has been developed as a biocompatible and biodegradable polymer for gene delivery, exhibiting high transfection efficiency with minimal toxicity [25]. The ability to design PUs that degrade in response to specific physiological conditions, such as pH changes, further enhances their applicability in gene therapy [13].

Our study achieved its objectives by demonstrating PU-controlled biodegradability through first-order degradation kinetics. The Higuchi diffusion model confirmed sustained DNA/RNA release, ensuring efficient gene delivery. Additionally, adsorption and internalization models showed saturation-dependent membrane attachment and progressive cellular uptake. These findings align with existing literature, reinforcing PU’s potential as a versatile gene delivery vector and providing a solid computational framework for optimizing its therapeutic efficiency.

Studies have further explored the application of PU vectors beyond cancer therapy, including cardiovascular diseases and genetic disorders. For example, PU carriers have been used to deliver genes encoding vascular endothelial growth factor (VEGF). They show promise in promoting angiogenesis in ischemic tissues, underscoring their potential in regenerative medicine [25,31]. PU-based systems for delivering small interfering RNA (siRNA) have also opened new possibilities for gene silencing therapies, particularly effective against diseases caused by abnormal gene expression [13,14]. Furthermore, PU nanomicelles have been developed to deliver chemotherapeutic agents, such as paclitaxel, improving the efficacy of cancer treatments while reducing systemic toxicity [36,37]. Moreover, the regulatory landscape for gene therapy and drug delivery products is complex, necessitating thorough characterization and validation of the safety and efficacy of PU-based delivery systems before they can be approved for use in humans [13,14].

In oncology, PU can deliver microRNA (miRNA) or small interfering RNA (siRNA), inhibiting gene expression in tumor growth. This could enhance the effectiveness of targeted treatments and reduce the side effects associated with chemotherapy. Additionally, delivering genes that encode pro-apoptotic proteins could trigger the death of cancer cells without affecting healthy tissues [38].

In genetic diseases, PU can serve as a vector for the delivery of recombinant DNA, essential for gene replacement therapies. For example, cystic fibrosis treatments could transport genes encoding the functional CFTR protein, thereby correcting genetic defects that lead to mucus buildup in the lungs [39].

PU could facilitate the delivery of genes that produce neuroprotective factors in neurodegenerative diseases, slowing neuronal degeneration. Lastly, PU holds potential in immunotherapy, assisting in the delivery of genes encoding tumor-specific antigens, thereby stimulating the immune response in cancer therapies. Furthermore, it could contribute to developing genetic vaccines, where DNA or messenger RNA fragments are delivered to train the immune system to recognize pathogens or cancer cells [40].

A significant limitation of this research is its entirely theoretical approach, which relies on mathematical modeling and numerical simulations without experimental confirmation. Although computational methods offer valuable insights into the behavior of PU-based gene delivery systems, the lack of in vitro and in vivo validation restricts the ability to verify the accuracy of the predicted outcomes in biological settings. Additionally, the models depend on various assumptions related to degradation kinetics, diffusion processes, and cellular uptake, which may not fully reflect the complexity of fundamental biological interactions. Another challenge is the scarcity of extensive experimental data on PU vectors, making it difficult to validate the parameters used in the simulations. Moreover, while this study explores the fundamental mechanisms of PU-mediated gene delivery, it does not consider potential immune responses, toxicity risks, or long-term stability issues that could impact its clinical application.

Future research on PU-based gene delivery should focus on enhancing biocompatibility, targeting efficiency, and controlled degradation while addressing key challenges in clinical translation. Functionalizing nanoparticles for receptor-mediated endocytosis can improve specificity, and stimuli-responsive PUs that degrade under physiological conditions may optimize gene release. Additionally, computational modeling and machine learning can refine polymer design and release kinetics, reducing the need for extensive experimental trials. However, despite these advancements, scalable production, reproducibility, and comprehensive safety evaluations remain significant hurdles for clinical application. The regulatory landscape for gene therapy requires rigorous characterization and validation of PU vectors before approval for human use. Overcoming these challenges will be crucial to unlocking their full potential in personalized medicine and offering improved treatments for cancer, neurodegenerative disorders, and genetic diseases.

## 5. Conclusions

This study demonstrates that PU exhibits first-order degradation kinetics, directly influencing the drug release duration. The encapsulated DNA/RNA is released exponentially over time, allowing for precise control over gene delivery. Furthermore, PU vectors effectively attach to the cellular membrane and are progressively internalized, with the process being limited by membrane saturation and intracellular degradation. These simulations confirm that PU can serve as an efficient vector for gene delivery. The optimization of its properties, guided by mathematical models, can significantly enhance the efficiency of transport and cellular uptake, paving the way for improved therapeutic outcomes.

## Data Availability

The data presented in this study are available upon request from the corresponding author.

## Abbreviations

The following abbreviations are used in this manuscript:

AuNPs	gold nanoparticles
CPPs	cell-penetrating peptides
DNA	deoxyribonucleic acid
FDA	U.S. Food and Drug Administration
MDPI	Multidisciplinary Digital Publishing Institute
MNPs	magnetic nanoparticles
PEI	polyethylenimine
PLL	poly(L-lysine)
PU	polyurethane
RNA	ribonucleic acid
VEGF	vascular endothelial growth factor
